# Imported cases of dengue in Rwanda: A call for One Health integrated surveillance and response system for arboviral diseases

**DOI:** 10.1016/j.ijregi.2026.100914

**Published:** 2026-05-12

**Authors:** Thierry Zawadi Muvunyi, Pierre Gashema, Emmanuel Kabalisa, Patrick Gad Iradukunda, Emmanuel Edwar Siddig, Isabelle Mukagatare, Jean de Dieu Harelimana, Mariska Jacoba Kreuger, Claude Mambo Muvunyi

**Affiliations:** 1 King Faisal Hospital, Kigali, Rwanda; 2 Africa Centres for Disease Control and Prevention (Africa CDC), Addis Ababa, Ethiopia; 3 Rwanda Biomedical Centre, Kigali, Rwanda; 4 Rwanda Food and Drugs Authority, Kigali, Rwanda

**Keywords:** Dengue, Rwanda, Diagnosis, Travelers

## Abstract

The true burden of dengue on the African continent is likely underestimated, with approximately 80% of confirmed cases originating from West Africa. In this report, we describe for the first time two imported mild dengue cases detected in travelers returning to Rwanda from Brazil and the Central African Republic. The first case involved a 58-year-old woman from Uganda traveling through Rwanda to Brazil, who presented with fever and lymphadenopathy on June 2, 2024. The second case was a 32-year-old man from Kicukiro District, who sought medical care on September 26, 2024 and exhibited fever and general weakness after visiting the Central African Republic. The traveler from Brazil was infected with dengue virus (DENV)-1, whereas the traveler from Central Africa carried DENV-2. This report underscores the growing risk of dengue being introduced into Rwanda through international travel and highlights the critical need to establish robust cross-border surveillance systems. It calls for improved early detection, active vector monitoring, and the enhancement of national and regional response strategies in line with the International Health Regulations.

## Highlights

• Rwanda has reported its first cases of dengue fever.
• Both cases involved international travelers.
• There is an urgent need to enhance active surveillance and early detection systems.
• Strengthening local laboratory capacity is essential to improve disease surveillance in Rwanda.
• Supporting dengue monitoring is vital for effective outbreak preparedness and response in Rwanda.

## Introduction

Dengue is a vector-borne viral infection that is caused by one of four closely related dengue viruses, dengue virus (DENV) 1-4. It continues to be a significant public health concern worldwide, with approximately 14 million reported cases and more than 10,000 deaths attributed to the disease each year [1].

The disease was primarily circulating in the tropical and subtropical regions where environmental conditions are conducive to the proliferation of *Aedes* mosquitoes, the primary vectors responsible for transmission [2,3]. However, climate change and the increased number and size of intentionally or accidentally created manmade bodies of water have created an environment suitable for the vectors. In Africa, the prevalence of dengue is rapidly growing, spanning countries such as Burkina Faso, Cameroon, Cabo Verde, Central African Republic, Chad, Côte d’Ivoire, Ethiopia, Ghana, Guinea, Kenya, Mali, Mauritius, Niger, Nigeria, São Tomé and Príncipe, Senegal, Sudan, and Togo [1]. Notably, until now, Rwanda had not reported any cases of dengue fever. Rwanda experiences increased international travel and trade; the risk of imported infectious diseases, including dengue, has become more imminent. This letter documents and discusses the first two imported cases of dengue in Rwanda.

## Case presentation

The first case involved a 58-year-old Ugandan female who lived in Brazil with her family and came to Rwanda for spending for a holiday; the patient presented with fever, headache, generalized body pain, vomiting, and fatigue on June 2, 2024. She had recently traveled from her country of origin, Brazil. Her baseline laboratory results were as following: hemoglobin = 12.7 g/dl, white blood cell count = 1900, platelets = 266,000; and her renal and liver function test were within the normal value, except it showed an elevated level of lactate dehydrogenase of 210 IU/L. The second case was that of a 32-year-old male resident of Kicukiro District, who sought medical care on September 26, 2024, with fever and general body weakness after a trip to the Central African Republic. Both individuals were diagnosed with dengue fever through real-time polymerase chain reaction testing at the National Reference Laboratory. Interestingly, the traveler from Brazil was infected with DENV-1, whereas the traveler from the Central African Republic was infected with DENV-2. The patients received supporting treatment that includes antibiotics, vitamin C, and antipyretic (paracetamol) and had fully recovered.

## Discussion

These cases highlight the role of international travelers in the spread of infectious diseases including dengue. Dengue was introduced into Africa from other countries; for instance, in Sudan, dengue was introduced in West Sudan for the first time in 2014 through the United Nations Peacekeeping Forces that were deployed from dengue-endemic countries to restore peace in Darfur region [4]. Similarly, dengue was detected among Australian solders returning from mission in endemic countries [5]. Notably, these cases also represent the first-ever reported instances of dengue in Rwanda.

Recognizing the increasing risk of climate-sensitive diseases, particularly, vector-borne ones such as dengue, countries worldwide should strengthen their surveillance and control measures for disease vectors, with a specific focus on invasive species. Early detection and rapid response are crucial for preventing the local establishment of these emerging diseases. This response must be strengthened through enhanced cross-country coordination and collaboration in surveillance efforts [6,7]. In addition, investment in diagnostic and surveillance capabilities is vital to enable timely identification of infections. Implementing molecular and genomically enhanced xenosurveillance could provide an effective and cost-efficient solution for the early detection of invasive disease vectors and emerging pathogens [7,8].

In addition, dengue diagnosis presents significant challenges primarily due to its symptom overlap with other infectious diseases, notably, malaria [9,10]. This similarity in clinical presentation can lead to delays in accurate diagnosis, which may subsequently result in worsened health outcomes for patients, especially those co-infected with multiple pathogens. The complexity of distinguishing dengue from other febrile illnesses complicates timely intervention and appropriate management [10].

In Rwanda, dengue surveillance efforts are currently fragmented and lack cohesion, which hampers a comprehensive understanding of the disease’s epidemiology within the country [11,12]. There is a notable scarcity of detailed data concerning the different serotypes circulating locally, the ecology of the mosquito vectors responsible for transmission, and the true mortality rates associated with dengue infections. This deficiency underscores the urgent need for expanded research initiatives aimed at filling these knowledge gaps. Better data collection and analysis would enhance the understanding of dengue’s regional dynamics, improve diagnostic accuracy, and inform targeted public health strategies to control and prevent outbreaks effectively. Therefore, we call for enhancing the implementation of a cross-borders surveillance and control for disease vectors and vector-borne diseases as part of the International Health Regulations (2005). This should be supported with the timely reporting and public sharing of public health information. Countries at risk of vector-borne diseases should consider implementing xenosurveillance for monitoring the dynamics of diseases vectors and pathogens. More investment is needed from the global community to support the research and development of novel prevention and control tools including safe and cost-effective vaccines and case management regimens.

In conclusion, there is an urgent need to enhance active surveillance and early detection systems in Rwanda. Strengthening local laboratory capacity is essential to improve disease surveillance efforts while supporting dengue monitoring is vital for effective outbreak preparedness and response. These measures will significantly contribute to public health outcomes in the region.

## Declaration of competing interest

The authors have no competing interests to declare.
